# *Candida palmioleophila*: A New Emerging Threat in Brazil?

**DOI:** 10.3390/jof9070770

**Published:** 2023-07-21

**Authors:** Gisela Lara da Costa, Melyssa Negri, Rodrigo Prado Rodrigues de Miranda, Danielly Corrêa-Moreira, Tatiana Castro Abreu Pinto, Livia de Souza Ramos, Deisiany Gomes Ferreira, Bruna Salomão, Tulio Machado Fumian, Camille Ferreira Mannarino, Tatiana Prado, Marise Pereira Miagostovich, André Luis Souza dos Santos, Manoel Marques Evangelista Oliveira

**Affiliations:** 1 Laboratory of Taxonomy, Biochemistry and Bioprospecting of Fungi, Oswaldo Cruz Institution (IOC), Oswaldo Cruz Foundation (Fiocruz), Rio de Janeiro 21040-900, Brazildcorrea@ioc.fiocruz.br (D.C.-M.); 2 Medical Mycology Laboratory, Clinical Analysis Department, State University of Maringá, Maringá 87020-900, Brazil; 3 Insect Biochemistry and Physiology Laboratory, Oswaldo Cruz Institution (IOC), Oswaldo Cruz Foundation (Fiocruz), Rio de Janeiro 21040-900, Brazil; 4 Laboratory of Pathogenic Cocci and Microbiota, Paulo de Goés Institute of Microbiology, Federal University of Rio de Janeiro, Rio de Janeiro 21941-853, Brazil; 5 Laboratory for Advanced Studies of Emerging and Resistant Microorganisms, Federal University of Rio de Janeiro, Rio de Janeiro 21941-853, Brazilandre@micro.ufrj.br (A.L.S.d.S.); 6 Laboratory of Microbiology, Federal Hospital of Andaraí, Rio de Janeiro 20541-170, Brazil; brunagssalomao@gmail.com; 7 Laboratory of Comparative and Environmental Virology, Oswaldo Cruz Institute, Oswaldo Cruz Foundation, Rio de Janeiro 21040-360, Brazil

**Keywords:** *Candida palmioleophila*, Brazil, emerging pathogen, MALDI-TOF MS

## Abstract

Human activity directly or indirectly causes climate change, promoting changes in the composition of the atmosphere. This change is beyond the variation of the natural climate. In this manner, climate change could create an environmental pressure which is enough to trigger new fungal diseases. In addition to climate alterations, the onset of the COVID-19 pandemic has also been associated with the emergence of fungal pathogens. Fungi showed that an inability to grow at high temperatures limits the capacity of fungi to infect mammals. However, fungi can develop thermotolerance, gradually adapting to rising temperatures due to climate change, and generating a greater number of disease-causing organisms. In the present study, we reported the detection and identification of *Candida palmioleophila* isolates recovered from raw sewage samples in Niteroi city, Rio de Janeiro State, Brazil, during a monitoring program for measuring SARS-CoV-2 presence and concentration. Using polyphasic taxonomy to identify the species and evaluating some virulence aspects of this species, such as biofilm formation and extracellular enzyme production, our data highlight this species as a possible emerging pathogen in Brazil, especially in the pandemic context.

## 1. Introduction

According to the United Nations Framework Convention on Climate Change, human activity directly or indirectly causes climate change, promoting changes in the composition of the atmosphere. This change is beyond the variation of natural climate [1]. In this manner, climate change could create environmental pressure which is enough to trigger new fungal diseases [2]. In general, the inability to grow at high temperatures limits the capacity of fungi to infect mammals. However, fungi can develop thermotolerance, gradually adapting to rising temperatures due to climate change and generating a greater number of disease-causing organisms [3,4]. In addition to thermotolerance, climate change can expand the reach of pathogenic species or their vectors, leading to the emergence of new diseases in places where they have not been previously reported [3].

An example of a fungus that may have emerged due to the climate alterations is *Candida auris*, a yeast more thermotolerant than many other fungal species. Without knowledge of its natural habitat, it is not possible to accurately determine whether rising temperatures played a significant role in the emergence of *C. auris* as a human pathogen [5]. However, according to Casadevall et al. (2021) [6], *C. auris* was an environmental species before emerging as a pathogenic fungus to humans, being unable to survive in anaerobic environments, and it is more commonly found in the cooler areas of the skin rather than in the viscera of mammals.

In addition to climate alterations, the onset of the COVID-19 pandemic has also been associated with the emergence of fungal pathogens. Among those that deserve careful attention are certain rare *Candida* species and other pathogenic yeasts, including *Clavispora lusitaniae*, *Candida intermedia*, *Candida auris*, *Diutina rugosa*, *Wickerhamiella pararugosa*, *Yarrowia lipolytica*, *Pichia norvegensis*, *Aspergillus fumigatus*, *Candida nivariensis*, *Kluyveromyces marxianus*, *Wickerhamomyces anomalus*, *Candida palmioleophila*, *Meyerozyma guilliermondii*, *Meyerozyma caribbica* and *Debaryomyces hansenii*, which have been increasingly reported in candidemia cases [7].

*Candida palmioleophila* was first discovered in 1988 by Nakase et al., and it was later described in 1999 as an etiologic agent of catheter-related fungemia [8]. This yeast is included in the group of rare Saccharomycotina yeasts and, although they can present intrinsic resistance to antifungal agents, there is still a lack of knowledge of their antifungal susceptibility profile [9].

To date, only few cases of candidiasis from *C. palmioleophila* have been reported [10,11]. Datta and colleagues (2015) [12] reported the first case of endogenous endophthalmitis from *C. palmioleophila* in Denmark. *C. palmioleophila* can cause endogenous fungal endophthalmitis, which can lead to complications in 2 to 26% of candidemia cases [10]. Scapaticci et al. (2018) [13] were able to identify *C. palmioleophila* infection only when reviewing laboratory results at the Microbiology Unit of San Camillo Hospital of Treviso, Italy. This species has been notoriously confused with *Candida famata*, the last one being highly phenotypically like *Candida guilliermondii* [11]. In the study by Jensen and Arendrup (2011) [11], *C. palmioleophila* isolates showed a uniform growth pattern: optimal growth at a temperature of 40 °C, intense budding without pseudohyphae formation after 14 days and presentation of a color change on CHROMagar solid medium, from turquoise to a turquoise scintillant to pink (all clinical isolates).

In this sense, an accurate identification of fungal species with unique susceptibility profiles, such as *C. palmioleophila*, is necessary, as treatment strategies are generally guided by species identification [11]. Conventional identification methods require several biochemical and phenotypic tests, which can lead to extensive time lengths and yet insufficient precision for discriminating *C. palmioleophila* from other species [11]. In this scenario, MALDI-TOF MS, recently introduced in many clinical microbiology laboratories worldwide, has proved to be a fast and powerful alternative to accurately identify several species of yeasts and molds [11].

In the present study, we reported the detection and major characteristics of *C. palmioleophila* isolates recovered from raw sewage samples in Niteroi city, Rio de Janeiro State, Brazil, during a monitoring program for measuring SARS-CoV-2 presence and concentration.

## 2. Materials and Methods

Sample collection and processing: raw sewage samples were obtained during the weekly monitoring program for SARS-CoV-2 investigation in Niteroi city, Rio de Janeiro State, Brazil, as previously reported [14]. Samples were recovered during the first wave of COVID-19 from two wastewater treatment plants, two sewer pipes (SPs) in surrounding neighborhoods and three SPs in slum communities, using sterile polypropylene bottles. Sewage samples were pasteurized at 60 °C for 90 min for virus’ inactivation, as recommended by Wu et al. (2020) [15]. Viral loads in these samples ranged from 3.67 to 4.59 log10, with median value of 4.12 log10 GC/100 mL and mean value of 4.15 ± 0.41 SD (standard deviation) [14].

For yeast isolation, 45 mL of each pasteurized sewage sample was centrifuged at 4000× rpm for 5 min, and the pellet resuspended in 250 µL of saline. Then, 20 µL of this solution was inoculated onto Sabouraud Dextrose Agar (SDA) plates (Difco, Becton-Dickinson and Company, Franklin Lakes, NJ, USA) with 400 mg/L of chloramphenicol and 25 mg/L of gentamicin and incubated at 35 °C for up to 5 days.

Phenotypic characterization: colonies grown on SDA (Difco, Becton-Dickinson and Company, Franklin Lakes, NJ, USA) were inoculated onto CHROMagar^®^ Candida (Difco, Becton-Dickinson and Company) and CHROMagar Candida PlusTM (CHROMagar, Paris, France) plates, which were incubated at 37 °C for 48 h. Interpretation of results was based on the manufacturer’s guidelines. Colonies presenting colors that were different from those expected, including *C. albicans* (light green), *C. tropicalis* (blue) and *C. krusei* (light pink) in CHROMagar^®^ Candida, were selected for further identification tests. Metabolic properties, such as sugar assimilation and enzymatic reactions, were analyzed by VITEK 2 system (bioMerieux, Craponne, France) using YST card, according to the manufacturer’s guidelines.

Molecular identification: genomic DNA was extracted using the Gentra^®^ Puregene^®^ Yeast and G+ Bacteria Kit (Qiagen^®^, Germantown, MD, USA), according to the manufacturer’s instructions. For PCR analysis, each isolate was tested with the universal fungal primers ITS1 (CGTAGGTGAACCTG CGG) and ITS4 (TCCTCCGCTTATTGATATGC) [16], which target the ITS1-5.8S-ITS2 region of the rDNA gene.

Amplification products were purified with the QIAquick^®^ PCR Purification Kit (QIAGEN^®^), according to the manufacturer’s protocol. Automated sequencing was carried out using the Sequencing Platform at Fundação Oswaldo Cruz—PDTIS/FIOCRUZ, Brazil. Nucleotide sequences were edited using the CodonCode Aligner software and compared using BLAST (Basic Local Alignment Search Tool) with sequences available at NCBI/GenBank database.

Phylogenetic analysis was carried out using the neighbor-joining algorithm of Saitou and Nei [17], with 1000 replicate bootstraps, based on alignment of the obtained ITS sequences of typed reference strain CBS7418 (MK394112.1) deposited in GenBank.

MALDI-TOF MS identification: isolates were subjected to MALDI-TOF MS, following previously described instructions [18]. Briefly, yeast cells were treated with 70% formic acid and acetonitrile. Then, 1 µL of the resulting extract was spotted onto the MALDI-TOF MS stainless plate (MBT Biotarget 96 IV™, Bruker, Bremen, Germany) and covered with 1 µL matrix solution α-cyano-4-hydroxycinnamic acid (CHCA, Fluka, Buchs, Switzerland). Each sample was analyzed in triplicate. The sample was air dried at room temperature before the spectra acquisition in Microflex mass spectrometer (Bruker Daltonics, Bremen, Germany) using Flexcontrol, version 3.0, and spectra were imported and analyzed using Maldi Biotyper (version 2.0; Bruker Daltonics, Bremen, Germany). Results were expressed as score values ranging from 0 to 3, where values of ≥1.7 are generally used for reliable genus identification and score values of ≥2.0 are used for reliable species identification.

Biofilm formation: fungal cell suspensions in Sabouraud broth (200 µL containing 10^6^ cells) were transferred into each well of a flat-bottom 96-well polystyrene microtiter plate and then incubated without agitation at 37 °C for 48 h. Medium-only blanks were also set up in parallel. Subsequently, the supernatant fluids were carefully removed, and the wells were washed three times with PBS to remove non-adherent fungal cells. Biomass quantification was assessed as described by Peeters and co-worker (2008) [19]. The biofilms were fixed with 200 μL of 99% methanol for 15 min and the supernatants were then discarded. Microtiter plates were air-dried for 5 min and then 200 μL of 0.4% crystal violet solution (stock solution diluted in PBS; Sigma-Aldrich, St Louis, MO, USA) was added to each well and the plates were incubated at room temperature for 20 min. The wells were washed once with PBS to remove excess stain, and the biomass in each well was then decolorized with 200 μL of 33% acetic acid for 5 min. One hundred microliters of the acetic acid solution were transferred to a new 96-well plate and the absorbance measured at 590 nm, using a microplate reader (SpectraMax M3; Molecular Devices, Sunnyvale, CA, USA).

Production of hydrolytic enzymes: the production of aspartic protease, phospholipase, esterase and phytase was evaluated by means of agar plate assays, as described by Price et al., 1982 [20]. The aspartic protease activity was determined according to Rüchel et al., 1982 [21], with modifications, using 1.17% yeast carbon base medium supplemented with 1% bovine serum albumin (BSA). Phospholipase activity was determined using the egg yolk agar plate (Sabouraud dextrose agar supplemented with 1 M NaCl, 5 mM CaCl_2_ and 2% sterile egg yolk emulsion, pH 7.0) described by Price et al. (1982) [21], with modifications. The esterase production was assayed using the Tween agar plate (peptone, 10 g; NaCl, 5 g; CaCl_2_, 0.1 g; agar, 1.5%; Tween, 0.1%; and pH 7.0 in 1000 mL of distilled water) according to Aktas et al. (2002) [22], with modifications. Phytase activity was evaluated using the calcium phytate agar (glucose, 10 g; (NH_4_)_2_SO_4_, 0.5 g; KCl, 0.2 g; MgSO_4_·7H_2_O, 0.1 g; calcium phytate, 2 g; yeast extract, 0.5 g; MnSO_4_, 0.005 g; FeSO_4_, 0.005 g; agar, 15 g; pH 7.0 in 1000 mL of distilled water), according to Tsang (2011) [23], with modifications. Aliquots (10 µL) of 48 h old cultured fungal cells (10^7^ cells/mL) were spotted on the surface of each agar medium and incubated at 37 °C for up to 7 days. The colony diameter (a) and the diameter of the colony plus the hydrolysis/precipitation zone (b) were measured using a digital paquimeter, and the production of each hydrolytic enzyme was expressed as Pz value (a/b), as previously described (Price et al., 1982) [20]. The Pz value was scored into four categories: Pz of 1.0 indicated no production; Pz between 0.999 and 0.700 indicated weak producers; Pz between 0.699 and 0.400 corresponded to good producers; and Pz lower than 0.399 meant excellent producers (Price et al., 1982) [20].

Antifungal susceptibility assay: the in vitro susceptibility was assessed using two methodologies: the Vitek 2 system (bioMerieux, France) and minimum inhibitory concentration (MIC), based on the Clinical and Laboratory Standards Institute protocol M27-A2 (CLSI, 2002) [24]. The Vitek 2 system (bioMerieux, France) included the evaluation of the following antifungals and drug concentrations: 1 to 32 µg/mL of amphotericin B, 1 to 8 µg/mL of caspofungin, 1 to 16 µg/mL of fluconazole, and 0.5 to 8 µg/mL of voriconazole. The AST-YS06 Vitek 2 card did not contain the antifungal itraconazole.

Susceptibility to amphotericin B and fluconazole was determined by minimum inhibitory concentration (MIC) based on the Clinical and Laboratory Standards Institute protocol M27-A2 (CLSI, 2002) [24] for all isolates, using the broth microdilution method. The concentrations tested were 16 μg/mL to 0.03 μg/mL for amphotericin B (Sigma-Aldrich, Brazil) and 64 μg/mL to 0.125 μg/mL for fluconazole (Pfizer, Itapevi, SP, Brazil). Candida albicans ATCC 90,028 was used as quality control. The test was performed in Roswell Park Memorial Institute 1640 medium, with L-glutamine, without sodium bicarbonate and 2% glucose (RPMI Medium 1640; Gibco, Grand Island, NY, USA), buffered (pH 7.05) with 0.165 M 3-(N-morpholino) propanesulfonic acid (Sigma–Aldrich, St. Louis, USA). After incubation at 37 °C for 48 h, MIC was determined by direct observation, according to CLSI guidelines M27-A2. MIC was defined as the lowest concentration of the antifungal agent that was able to inhibit the growth of 50% and/or 90% of the population compared to the drug-free control. The reading of microplates was carried out at 530 nm using the Expert Plus Microplate Reader (ASYS). Results were interpreted according to CLSI supplements M27-S3 and M27-S4.

Infection of *Tenebrio molitor* larvae assay: survival curve determination with infected larvae was performed according to Jarros et al. [25], with modifications. To ensure the reliability of the results, all isolates were grown under the same conditions (time, temperature, under agitation and in the same culture medium), and the larvae selected for the study were in the same period of development, with equivalent size and weight, and were inoculated with the same cell concentration. Briefly, larvae were selected weighing between 100 and 200 mg with light and uniform color, no dark spots or grayish markings, and having been previously separated into their respective infection groups. The larvae were separated into groups of 10 larvae each, and divided into negative the control group, the group without inoculum, the positive control group, the group inoculated with PBS, and one group for each fungal isolate. 

The isolates were grown at 25 °C under 130 rpm agitation for 18 h in Sabouraud dextrose broth (SDB; Kasvi, Spain). The cultures were centrifuged, the supernatant discarded, and they were washed three times with phosphate-buffered saline (PBS) at 8000 rpm for 5 min at 4 °C. After washing, the inoculum concentration in the Neubauer chamber was adjusted to 1 × 10^5^/10 μL in PBS for infection. 

For inoculation, each larva received 10 μL of the respective inoculum, using an insulin syringe (1 mL capacity). Inoculation was in the hemocele, between the second and third sternite visible above the legs, in the ventral portion. After infections, the larvae were kept in disposable Petri dishes containing rearing diet, at 25 °C. The number of larvae was recorded at 24 h intervals for 10 days. To establish the death of the larvae, melanization and lack of response to physical stimuli were visually analyzed, with melanization verified from a touch with surgical tweezers.

## 3. Results

**Figure 1:**
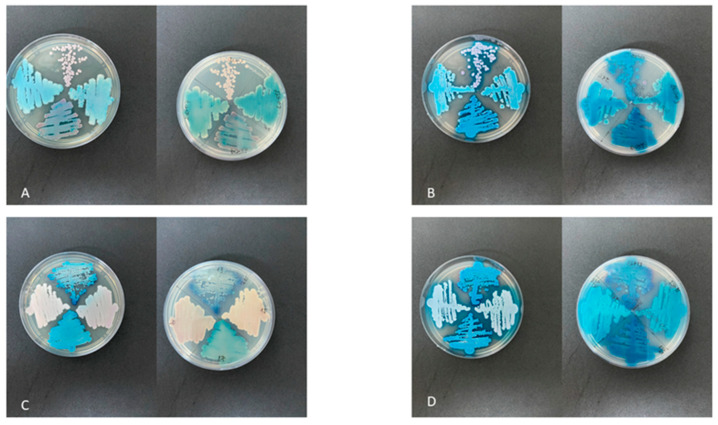
(**A**,**C**)—Growth of isolates of *Candida palmioleophila* in BD^TM^ CHROMagar^TM^ Candida Medium (BD Difco) incubated at 37 °C for 48 h. (**B**,**D**) Growth of isolates of *Candida palmioleophila* in CHROMagar Candida Plus (CHROMagar^TM^) incubated at 37 °C for 48 h.

Isolation and identification of yeast species: seven yeast isolates were obtained from the sewage samples, one from each sample. Only one color pattern was detected in each plate, demonstrating that there were no mixed colonies in each sample (Figure 1A–D).

Except for the expected colony colors for *C. albicans* (light green), *C. tropicalis* (blue) and *C. krusei* (light pink), only two other color standards were observed in the chromogenic medium: turquoise or rose and white (Figure 1A–D). According to Jensen and Arendrup (2011) [14], turquoise or rose colonies indicate *C. palmiolephila* species, while white colonies indicate *Candida* sp., according to manufacturer guidelines.

**Figure 2:**
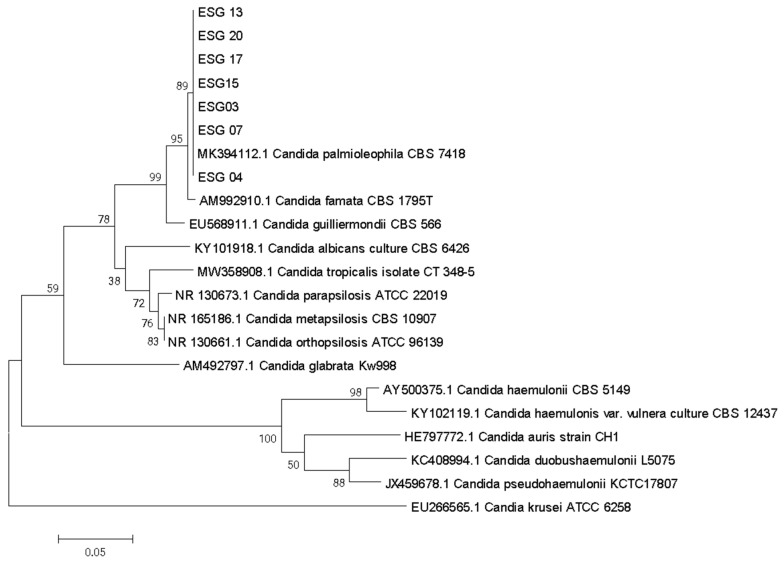
Phylogenetic relationships among the isolates of the clinical sample with reference strains of the *Candida* spp. inferred from *ITS* sequences using the neighbor joining method [17]. The optimal tree is shown. The percentages of replicate trees in which the associated taxa are clustered together in the bootstrap test (1000 replicates) are shown next to the branches. The evolutionary distances were computed using the maximum composite likelihood method and are in the units of the number of base substitutions per site. This analysis involved 22 nucleotide sequences. There was a total of 238 positions in the final dataset. Evolutionary analyses were conducted in MEGA X.

In the VITEK 2 system, the average probability of biochemical identification of isolates varied from 33 to 94% (Table 1), these isolates being identified as *Candida albicans, Candida famata*, *Candida parapsilosis*, *Candida tropicalis* or *Kodameae ohmeri*, but none identified as *C. palmioleophila*. Sequencing of *ITS* region revealed that all seven isolates were *C. palmioleophila*, when compared to available *ITS* sequences from the NCBI/GenBank database (*C. palmioleophila* JN091166.1 and NR077076.1). (Figure 2).

The sequences of the isolates have been deposited in GenBank under the following accession numbers: OP428765 to OP728771. Likewise, all seven isolates were identified by MALDI-TOF MS as *C. palmioleophila* with scores ≥1.7 (Table 1). 

Antifungal susceptibility profile: results of antifungal susceptibility testing are shown in Table 1. Three strains (ESG04, ESG15 and ESG20) were not evaluated as to antifungal susceptibility in ViteK 2, due to the incorrect identification by the system. Among the remaining four isolates, all were susceptible to voriconazole (VRC), micafungin (MCF), amphotericin B (AMB), caspofungin (CSP) and flucytosine (FLC), while two showed resistances to fluconazole (FLU). Using the broth microdilution method, all seven isolates were susceptible to amphotericin B, with MIC90 of 1 µg/mL; however, five isolates were SDD or resistant to fluconazole, with MIC50 of 16 µg/mL and MIC90 of 32 µg/mL (Table 2).

Biofilm formation and production of extracellular enzymes: all seven isolates were able to produce biofilm after 48 h of incubation at 37 °C at different degrees, with absorbance values ranging from 0.239 to 1.008. We observed that, with exception of one isolate that exhibited no activity, all the remaining six isolates were good producers of esterase (85.7%), with Pz values ranging from 0.55 to 0.64. Two isolates (28.6%) were good producers of aspartic proteases (Pz 0.52 and 0.56) and phytase (Pz 0.59 and 0.67). On the other hand, none of the isolates were able to produce phospholipase activity (Table 3).

**Figure 3:**
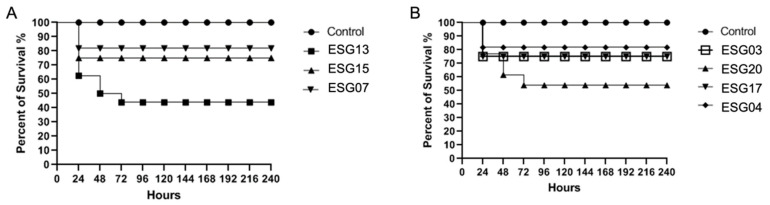
Survival curves of infected *Tenebrio molitor* with *Candida palmioleophila* isolates. Groups of 10 larvae were infected with each isolate of *C. palmioleophila*. (**A**) Samples ESG13, ESG15 and ESG 07; (**B**) Samples ESG03, ESG20, ESG 17 and ESG04. The negative control group of the *T. molitor* larvae was injected just with PBS (without yeasts).

Survival of *Tenebrio molitor* larvae: using the in vivo *Tenebrio molitor* larvae model, all seven isolates were able to cause larval death after 24 h of infection (Figure 3). After 24 h of infection, isolates ESG07 and ESG04 (Figure 3A,B) caused death in only 20% of the larvae. The isolates ESG15, ESG03, and ESG17 caused death in 30% of the larvae, and remained in this condition until the end of the experiment for these isolates. However, after 72 h, isolate ESG20 (Figure 3B) caused more than 50% of larval death, and ESG13 was the isolate that caused the most larval death, with more than 60% of death after 24 h of infection (Figure 3A).

## 4. Discussion

In our study, we successfully used a method for screening *Candida* species associated with the environmental samples in the presence of SARS-CoV-2 concentrations from wastewater samples obtained from different areas of Niterói city, Rio de Janeiro state, Brazil. Applying the biosafety measures previously recommended by Wu et al. (2020) [15] and Prado and collaborators (2021) [14], the wastewater samples were thermally inactivated prior to the integral inactivation of viral particles. The process of pasteurization of the sewage at 60 °C for 90 min to inactivate the virus ensured biosafety in the selection of the fungal samples, which were later identified as *Candida species* with a profile of resistance to high temperatures. There are studies in the literature that have documented the occurrence of yeasts at high temperatures, including during pasteurization processes, including those belonging to the *Candida* genus. Tsang and Ingledew [26] described the thermal resistance in several yeast species, including *Candida mycoderma*. Cell death kinetics studies followed by thermal kinetics assays indicated the tolerance and maintenance of cell viability of *C. mycoderma* propagules within a temperature range of 45 °C to 51 °C, revealing a certain profile of thermal resistance [26].

A similar thermal resistance profile was observed for the species *C. apicola*, where thermal kinetics assays showed the temporal decrease in yeast propagules exposed to a temperature range of 45 °C to 60 °C. At the maximum temperature applied in the assays, there were remaining propagules from the cell count, indicating relatively high thermal resistance for the aforementioned species [27].

Since the start of COVID-19 pandemic, several studies have demonstrated that wastewater-based epidemiology (WBE) is a useful tool for monitoring SARS-CoV-2 spread in some regions [14,28,29,30]. Nevertheless, there are no studies reporting the screening of *Candida* species associated with environmental samples of raw sewage, along with tolerance to high temperatures, as a resource for inactivation of samples at 65 °C. Thermic resistance can be an optimizing factor for the outbreak of minor pathogens in global populational phenomena such as migratory movements.

*Candida palmioleophila* yeast bodies have been found to be associated with wild populations of the penguin *Spheniscus magellanicus* [31]. Such interaction in the host-pathogen interface elucidated by the authors indicates a pathogenic species highly resistant to low temperatures and high osmotic levels. *C. palmioleophila* strain JKS4 was prospected as a bioremediation agent for the degradation of artificial azo dyes [32]. The physiological capabilities of the species were measured, such as capacity of detoxification under saline conditions. However, the strain JKS4 was able to optimally decolorize the azo dye RB5 in the presence of a sodium chloride concentration range from 0 to 5% (*w*/*v*) into a proper liquid medium. In contrast, different strains of *C. palmioleophila* were detected, composed mainly of the microbial community of a marine Centrolophidae species, *Seriolella violaceae*, maintained under laboratory conditions [33]. Previously, a wild strain of *C. palmioleophila* was isolated from marine ecosystems (such as swamps and sediment from continental platforms) and identified through DNA barcoding [34].

In Brazil, the occurrence of *C. palmioleophila* has been reported between the years of 2000 and 2001 in the hydrographic basin of Rio Doce, Minas Gerais, Brazil, mainly during dry seasons [35]. However, the ecological or physiological aspects of this species under tropical forest conditions are not known. Nevertheless, osmotic/thermal resistance is a common feature in certain *Candida* species. The *Candida albicans* complex is well studied for its osmotic adaptive metabolism [36,37,38,39]. Therefore, the detection of *C. palmioleophila* in wastewater in Brazil may represent a future concern for public health conjectures, since wastewaters are profusely discharged, even without treatment, iton different aquatic environments in Brazil, such as beaches and rivers.

In Italy, Pierantoni and collaborators (2020) [40] reported two cases of candidemia due to *Candida palmioleophila*, but they were misidentified as *Candida albicans* by using the Vitek2 system and CHROMagar *Candida* in the initial diagnosis. From the identification perspective, CHROMagar *Candida* medium can be misleading, especially when dealing with new emerging species. Indeed, CHROMagar^®^ *Candida* and CHROMagar^®^ *Candida* Plus do not represent a viable resource for identification of some species, according to the manufacturer’s guidelines. Here, *C. palmioleophila* isolates were identified as *Candida tropicalis*, *Candida parapsilosis* or *Kodamaea ohmeri* using the Vitek 2 system. Likewise, previous studies [14,40] reported biochemical identification as an inaccurate approach for the identification of *C. palmioleophila*. As an emerging species in European countries such as Denmark [41], *C. palmioleophila* is frequently misidentified as other *Candida* species such as *C. famata* and *C. guilliermondii* by the employment of traditional methods like chromogenic media, the dye pour-plate auxanogram, germ tube tests, or the Wickerham medium method [11,42].

In order to avoid misidentification, methods involving mass spectrometry attached to matrix-assisted laser desorption-ionization (MALDI-TOF), in addition to molecular and computational approaches, may be able to more accurately discriminate among highly correlated yeast species. MALDI-TOF MS has been exploited for its capacity to identify fungal species belonging to different fungal genera [43,44,45]. In our study, MALDI-TOF MS was 100% congruent with the partial sequencing of ITS region, reinforcing the ability of this technique to accurately identify fungal species, including the rarest ones.

Drug resistance in *Candida* species may be manifested phenotypically through diverse pathways in the metabolic, enzymatic and structural biology of yeasts. The upregulation of efflux pumps for the detoxification of nocive substances, points out mutations which results in the alteration of cell wall components such as beta-1,3-glucans (the main target for echinocandins’ mode of action); substantial changes in the ergosterol synthesis metabolic pathway and biofilm secretion may be mentioned as resistance mechanisms profusely described throughout the literature [46,47,48]. *Candida palmioleophila* shows a particular profile of susceptibility to fungicidal drugs such as echinocandins (anidulafungin and micafungin), in contrast to a fluconazole-resistant phenotype [14]. Different studies highlight azole resistance among clinically relevant fungal species. The loss of susceptibility to azole fungicides among Saccharomycotina species is associated with multifactorial circumstances, such as indiscriminate exposition to azoles, patient profiles, geographic localization and genetic particularities [49,50].

In our study, only two isolates (ESG03 and ESG17) showed a characteristic MIC indicating resistance to fluconazole. Previous studies [12,14] reported intrinsic fluconazole resistance in *C. palmioleophila* isolates. Stavrou et al. (2020) [9] reported increasing MIC values not only for fluconazole, the most-used antifungal drug, but also against other azoles, echinocandins and a small percentage of strains also showing multidrug resistance. Although only seven isolates were evaluated in this study, our results suggest that fluconazole may still be a treatment option for *C. palmioleophila* infection cases in Brazil.

Pathogenic and opportunistic fungi can harbor an arsenal of virulence attributes that allow them to survive and cause infection in the hostile environment of the human body. The ability to form biofilm and to produce different classes of hydrolytic enzymes are well-known virulence factors involved in *Candida* spp. infections, including *C. albicans* and many non-*albicans Candida* species, but knowledge of *C. palmioleophila* virulence factors is very scarce. Corroborating our findings, Mroczyńska and coworkers [50] reported the ability of biofilm formation on the part of three isolates of *C. palmioleophila* and evaluated the production of hydrolytic enzymes by the same three isolates of *C. palmioleophila*; they demonstrated that all of them (100%) produced aspartic protease activity, one (33.3%) produced esterase activity and, in contrast to our findings, two (66.7%) isolates produced phospholipase activity.

Experimental models are largely used in fungal virulence studies. In this context, insect models have economic, logistic and ethical advantages over mammalian models, and allow high-efficiency testing on a large scale at low cost. The advantage of this alternative model is that *T. molitor* larvae can be maintained at temperatures between 25 °C and 37 °C. *T. molitor* have been used to study the pathogenesis of fungi like *C. neoformans* and *C. albicans*, showing the effectiveness of this host as an infection model [51]. In our study, all isolates were able to cause death in the larvae after 24 h of infection. However, the isolates showed differences in pathogenicity, being strain-dependent, corroborating the findings of Mroczyńska et al. [50], who also observed the pathogenic capacity of *C. palmioleophila* and the difference among the strains. These data highlight the importance of studies with emerging species, especially in a context of host immunocompromise, for greater knowledge and prevention of diseases related to these isolates [50].

Altogether, these observations suggest that production of different classes of hydrolytic enzymes by *C. palmioleophila* seems to be strain-dependent. The same authors reported that *C. palmioleophila* isolates exhibited moderate virulence using *Galleria mellonella* as a host model [51].

In conclusion, we report the detection of *C. palmioleophila*, from environmental samples with SARS-CoV2 detected, highlighting this species as a possible emerging pathogen in Brazil, especially in the pandemic context. In addition to resistance to antifungal drugs, we have shown that *C. palmioleophila* isolates can also produce important virulence attributes that aid in the establishment of the infectious process. Climatic alterations and the COVID-19 pandemic have been associated with an increasing number of new emerging fungal pathogens that need careful and accurate identification at species level. However, discrimination of certain species is only achieved by molecular methods, which are generally expensive and need specialized technicians. In this scenario, our data reinforce that MALDI-TOF MS is an alternative methodology that can be faster and more effective when compared to conventional phenotypic methods, and more cost-effective when compared to genetic sequencing for the identification of *C. palmioleophila* isolates.

## Figures and Tables

**Table 1:** Species identification by polyphasic taxonomy.

Sample	Biochemical Identification	Probability Identification	Chromoagar *Candida* 37 °C						Chromoagar *Candida* Plus 37 °C				MALDI-TOF MS	ITS (Parcial Sequencing)
	(VITEK 2 System)	VITEK2 (%)	LT	LPWH	LGWH	LTPH	TQ	WMWH	LBWH	LB	TPH	LTWH		
ESG03	*C. albicans/C. famata/C. parapsilosis*	*33*	0	0	1	0	0	0	1	0	0	0	*C. palmioleophila*	*C. palmioleophila*
ESG13	*C. tropicalis/C. parapsilosis*	*50*	0	0	0	0	1	0	0	0	1	0	*C. palmioleophila*	*C. palmioleophila*
ESG15	*C. famata*	*92*	0	0	0	0	0	1	0	1	0	0	*C. palmioleophila*	*C. palmioleophila*
ESG17	*C. parapsilosis*	*91*	1	0	0	0	0	0	0	0	0	1	*C. palmioleophila*	*C. palmioleophila*
ESG04	*Kodamaea ohmeri*	*94*	0	0	0	1	0	0	0	1	0	0	*C. palmioleophila*	*C. palmioleophila*
ESG20	*C. famata*	*88*	0	1	0	0	0	0	0	1	0	0	*C. palmioleophila*	*C. palmioleophila*
ESG07	*C. parapsilosis*	*88*	0	0	1	0	0	0	1	0	0	0	*C. palmioleophila*	*C. palmioleophila*

LT: Light turquoise; LPWH: Light pink with white halo; LGWH: Light green with white halo; LTPH: Light turquoise with pink halo; TQ: Turquoise; WMWH: White to mauve with white halo; LB: Light blue; TPH: Turquoise with pink halo; LTWH: Light turquoise with white halo.

**Table 2:** Evaluation of susceptibility to determine values (µg/mL) of minimum inhibitory concentration (MIC) by broth microdilution and Vitek 2 against antifungals and against isolates of *Candida palmioleophila*.

Samples	Amphotericin B		Fluconazole		Voriconazole		Caspofungin		Micafungin		Flucytosine	
	µg/mL (Cut-Off)		µg/mL (Cut-Off)		µg/mL (Cut-Off)		µg/mL (Cut-Off)		µg/mL (Cut-Off)		µg/mL (Cut-Off)	
	Vitek2	CLSI	Vitek2	CLSI	Vitek2	CLSI	Vitek2	CLSI	Vitek2	CLSI	Vitek2	CLSI
**ESG 03**	1 (S)	0.25 (S)	8 (R)	4 (S)	£ 0.12 (S)	***	0.25 (S)	***	£ 0.06 (S)	***	£ 1 (S)	***
**ESG 04**	***	0.5 (S)	***	16 (SDD)	***	***	***	***	***	***	***	***
**ESG 07**	1 (S)	0.25 (S)	2 (S)	16 (SDD)	£ 0.12 (S)	***	0.25 (S)	***	£ 0.06 (S)	***	£ 1 (S)	***
**ESG 13**	0.5 (S)	0.25 (S)	1 (S)	1 (S)	£ 0.12 (S)	***	£ 0.12 (S)	***	£ 0.06 (S)	***	£ 1 (S)	***
**ESG 15**	***	0.25 (S)	***	64 (R)	***	***	***	***	***	***	***	***
**ESG 17**	1 (S)	0.5 (S)	8 (R)	32 (SDD)	***	***	£ 0.12 (S)	***	0.25 (S)	***	£ 0.06 (S)	***
**ESG 20**	***	0.25 (S)	***	16 (SDD)	***	***	£ 0.12 (S)	***	0.25 (S)	***	£ 0.06 (S)	***
**MIC50**	**1**	**0.25**	**2**	**16**	**£ 0.12**	***	**0.25**	***	**£ 0.06**	***	**£ 1**	***
**MIC90**	**1**	**0.5**	**8**	**32**	**£ 0.12**	***	**0.25**	***	**£ 0.06**	***	**£ 1**	***
**MIC Range**	**0.5–1**	**0.25–0.5**	**44774**	**23377**	**£ 0.12**	***	**£ 0.12–0.25**	***	**£ 0.06**	***	**£ 1**	***

*** MIC not identified.

**Table 3:** Analysis of Biofilm formation and production of extracellular enzymes.

Samples	Biofim	Esterase	Aspartic Protease	Phytase	Phospholipase
ESG 03	0.448 ± 0.092	0.63 ± 0.02	1.00 ± 0.00	1.00 ± 0.00	1.00 ± 0.00
ESG 04	1.008 ± 0.156	1.00 ± 0.00	0.52 ± 0.09	0.67 ± 0.07	1.00 ± 0.00
ESG 07	0.449 ± 0.096	0.62 ± 0.01	1.00 ± 0.00	1.00 ± 0.00	1.00 ± 0.00
ESG 13	0.811 ± 0.133	0.64 ± 0.01	0.56 ± 0.02	0.59 ± 0.04	1.00 ± 0.00
ESG 15	0.409 ± 0.096	0.56 ± 0.01	1.00 ± 0.00	1.00 ± 0.00	1.00 ± 0.00
ESG 17	0.239 ± 0.051	0.55 ± 0.01	1.00 ± 0.00	1.00 ± 0.00	1.00 ± 0.00
ESG 20	0.248 ± 0.065	0.61 ± 0.01	1.00 ± 0.00	1.00 ± 0.00	1.00 ± 0.00

## Data Availability

The data that support the findings of this study are available from the corresponding author upon reasonable request.
